# A Case Report of Progressive Liver Failure Inappropriate to Decompensated Heart Failure Following Infection With COVID-19

**DOI:** 10.7759/cureus.10142

**Published:** 2020-08-30

**Authors:** Jalil Makarem, Nikoo Naghibi, Mohammad Taghi Beigmohammadi, Morteza Foroumandi, Maryam Mehrpooya

**Affiliations:** 1 Anesthesiology and Critical Care, Imam Khomeini Hospital Complex, Tehran University of Medical Sciences, Tehran, IRN; 2 Cardiology, Imam Khomeini Hospital Complex, Tehran University of Medical Sciences, Tehran, IRN

**Keywords:** covid-19, liver failure, heart failure, respiratory failure

## Abstract

This article is about a known case of heart failure presented with acute liver failure following a coronavirus disease-2019 (COVID-19) respiratory tract infection. The patient was admitted with encephalopathy and respiratory distress with a positive COVID-19 reverse transcription-polymerase chain reaction (RT-PCR) test. Elevated liver enzymes, severe coagulopathy, and hypoglycemia were apparent without any clinical or laboratory findings of sepsis, acute viral hepatitis, medicine related or drug-induced, or autoimmune-related acute liver failure. Supportive and therapeutic measures related to his cardiovascular, respiratory, and liver function were executed in the ICU. Unfortunately, the patient expired because of respiratory failure.

## Introduction

In December 2019, an outbreak of a novel coronavirus started in Wuhan, China. Respiratory involvement is the most common damage caused by coronavirus disease-2019 (COVID-19) infection that may lead to acute respiratory distress syndrome [1]. Liver involvement has been reported; however, there are several questions regarding the importance and severity of liver dysfunction [2]. Although in more severe cases the prevalence of derangement in liver enzymes and serum bilirubin levels was higher, significant coagulopathy or a fulminant presentation of liver failure has not been reported yet in association with COVID-19 pneumonia [3]. However, our patient was already a candidate for a heart transplant because of compensated heart failure; the liver dysfunction and patient's presentation were more severe than would be expected from the severity of the patient's heart failure. Thereafter, we reported an acute and intensified liver failure in a patient with known cardiomyopathy following infection with COVID-19. Written informed consent was obtained from the patient's wife for this publication. 

## Case presentation

A 52-year-old male, a known history of ischemic cardiomyopathy [ejection fraction (EF)=15%] with a compensated heart condition and a candidate for heart transplantation in the next few months, was brought to the emergency ward on 1st April, 2020 due to abdominal pain, altered mental orientation, agitation, and slight respiratory distress. He was admitted in the infectious disease ward, with a diagnosis of probable COVID-19 infection and appropriate supportive treatment. He did not have any history of chronic liver disease accompanied by normal liver function tests and normal coagulation profiles in previous visits. However, he had suffered from mild chronic kidney disease, with serum creatinine 2 mg/dL and estimated glomerular filtration rate (GFR) 44 cc/min. He had been taking amiodarone (200 mg daily), aldactone (25 mg daily), lasix (40 mg twice a day), mexiletine (100 mg twice a day), allopurinol (100 mg daily), and renal supplement nephrovit (daily).

**Figure 1:**
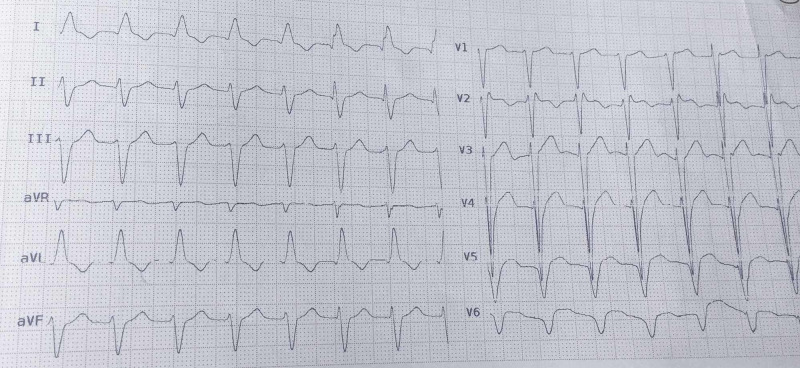
The patient's ECG. ECG showed normal sinus rhythm, left anterior hemiblock, and interventricular conduction delay. This ECG is compatible with advanced heart failure. ECG, electrocardiography

His vital signs at presentation were as follows: SPO2=87% ( without O2 supplement), pulse rate=108 beats per minute, respiratory rate=26 per minute, blood pressure=78/52 mmHg and in physical examination, coarse crackles in 1/3 both lungs, moderate respiratory distress, right and middle-upper abdominal tenderness and one plus pitting edema in lower extremities, were obvious. According to the primary clinical and laboratory evaluation, a fulminant liver failure associated with an acute kidney injury and a decompensated heart failure was diagnosed. The main related findings were highly elevated international normalized ratio (INR)=8, type A grade II encephalopathy based on The West Haven Criteria (WHC) (4), profound recurrent hypoglycemia, elevated liver enzyme [alanine transaminase (ALT)=325, aspartate aminotransferase (AST)=223] and increased serum bilirubin (total=4.4 and direct=2), blood urea nitrogen=86 as well as serum creatinine=2.1. Therefore, he was immediately transferred to the ICU. Electrocardiography of the patient is shown in Figure 1. 

**Figure 2:**
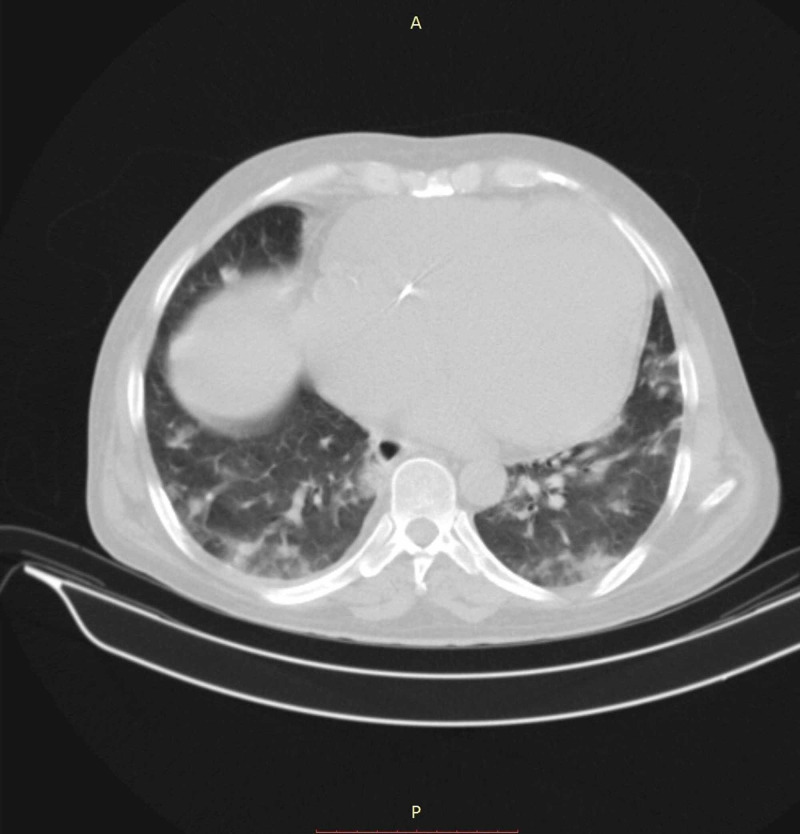
The patient's thoracic CT scan without contrast.

Bedside echocardiography showed moderate to severe left ventricular (LV) enlargement, very severe systolic dysfunction (EF=10%-15%), moderate right ventricular (RV) enlargement, tricuspid gradient: 30 mmHg, and pulmonary arterial pressure (PAP)=35 mmHg. Abdominal ultrasonography depicted normal echo of liver parenchyma, no liver mass or metastasis, sludge on the gallbladder, normal common bile duct and intrahepatic ducts, normal echo and diameter in both kidneys, no hydronephrosis but free fluid at inter-loop and hepatorenal area that was insufficient for tap. Chest CT scan showed multifocal, bilateral ground-glass opacities with peribronchovascular distribution, while an abdominal CT scan showed no significant abnormality (Figure 2).

 Supportive treatment included O2 therapy with reservoir mask, infusion of norepinephrine (10 mcg/min), furosemide infusion (5 mg/h), and milrinone infusion (0.2 mcg/kg/min). Based on radiologic findings compatible with COVID-19 pneumonia followed by a positive reverse transcription-polymerase chain reaction (RT-PCR) test, hydroxychloroquine (200 mg a day) and N-acetylcysteine (NAC) (1 g twice a day) were added.

 Because of oliguria, severe metabolic acidosis [pH=7.20, NaHCO3=13 meq/L, PaCO2=38 mmHg, base excess (BE)=-13] in addition to serum potassium=5.9 meq/L, a dialysis catheter was inserted in the right internal jugular vein under ultrasound guide, and three hours hemodialysis was performed subsequently. The patient's hemodynamic status, as well as oxygenation, were in a stable state.

Laboratory test results are shown in Tables 1-2. Severe coagulopathy and elevated liver enzyme were obvious. Erythrocyte sedimentation rate (ESR) and procalcitonin level were not significant. Two times blood culture and urine culture showed no significant growth. The results of the following tests were unremarkable: hepatitis A, B, C, Epstein Barr and Cytomegalovirus, and HIV. Tests for autoimmune markers were also negative. On the first day of ICU admission, serial blood glucose tests depicted profound hypoglycemia that was managed by an infusion of glucose 50% with control of glucose blood level.

**Table 1:** Serial laboratory findings. *After transfusion of five units of FFP; **After dialysis INR, International normalized ratio; AST, aspartate aminotransferase; ALT, alanine aminotransferase; ALP, alkaline phosphatase; ESR, erythrocyte sedimentation rate; CRP, C-reactive protein; FFP, fresh frozen plasma

Serial laboratory test	Follow up days					
	Day 1 (2^nd^ April)	Day 2	Day 3	Day 4	Day 5	Day 6
Hemoglobin (g/dL)	15.7	15.2	13.8	13.3	13.9	12.4
White blood cell count (∗10ˆ9/L)	9.1	22.8	18.7	7.2	10.2	9
Lymphocyte %	33.9	16	11	11.5	4.4	10.3
Platelet /µL *10^3	209	145	110	89	73	71
Serum sodium (meq/dL)	128	128	126	130	138	136
Serum potassium (meq/dL)	5.9	5.1	5.1	3.5	4.5	3.1
Blood urea nitrogen (mg/dL)	186	171	168	164	124	137
Serum creatinine (mg/dL)	3.1	6.9	4.1	3	1.7	1.5
Serum calcium (meq/dL)	8.8	8.1	7.1	6.9	8	9.8
Serum phosphorus (meq/dL)	2.6	3.8	5.5	4.3	3	5.4
Serum magnesium (meq/dL)	2.3	2.3	2.4	2	1.8	2.7
Prothrombin time (s)	>90	>90	>90/ 44.9*	>90	>90	52.9
Partial thromboplastin time (s)	86	96	>160/ 67*	96	>160	51
INR	>8	>8	>8/ 3.42*	>8	>8	4.04
The potential of hydrogen (pH )	7.21 / 7.33**	7.25	7.24	7.46	7.54	7.35
AST (U/L)	723	-	-	859	-	179
ALT (U/L)	825	-	-	910	-	301
ALP (U/L)	351	-	-	395	-	308
Total bilirubin (µmol/L)	4.4	-	-	6.4	-	16.4
Direct bilirubin (µmol/L)	2	-	-	3.6	-	9
ESR (mm/h)	5	-	-	10	-	9
CRP (mg/L)	45	-	-	52	-	56

 

 

**Table 2:** Result of laboratory tests. NT-pro BNP, N-terminal B-type natriuretic peptide

Laboratory test	Result
NT-pro BNP (pg/mL)	35000
Serum iron (µ/dL)	28
Ferritin (ng/mL)	1242
Amylase (U/L)	138
Lipase (U/L)	61
Uric acid (mg/dL)	2.3
Total protein (g/dL)	6.8
Serum albumin (g/dL)	3.2

Liver failure and heart failure and acute respiratory distress syndrome were not improved essentially. Decompensated respiratory function, which was associated with severe hypoxia and respiratory distress, accompanied by multi-organ dysfunction and unstable hemodynamic status, directed us to intubate the patient with an endotracheal tube on the sixth day of ICU admission. Therefore, the patient was supported by ventilator under mild sedation. On the next day, the patient died due to cardiac asystole nonresponsive to cardiopulmonary resuscitation.

## Discussion

In this study, we reported a severe acute liver failure simultaneously with COVID-19 infection presenting with respiratory and abdominal complaints in a patient with known heart failure.

The liver injury induced by heart failure was observed in 15%-65% of cases [4]. The main mechanisms of hepatopathy in association with heart failure included congestive hepatopathy due to elevated systemic venous pressure, inflammatory effects of heart failure on liver tissue and fibrosis induction, poor nutrition in patients with heart failure, and reduced cardiac output in conjunction with increased uric acid levels [5]. The mentioned evidence reminds us that the liver in these patients is more vulnerable to any insults than the normal population. However, our patient presented with no previous clinical or laboratory liver abnormality.

The incidence of hepatic abnormalities signiﬁcantly increases after COVID-19 infection and during the course of the disease, which may indicate the direct effect of severe acute respiratory syndrome-corona virus-2 (SARS-CoV-2) on the hepatocytes, and impacts of excessive immune system reactions on the liver or the side effects of the prescribed medications to the patient's liver [6]. The severity of the liver injury or liver function derangement has been reported from mild to modestly elevated liver enzymes, plasma bilirubin, mild coagulopathy, and slightly decreased serum albumin in COVID-19 infected patient. In more severe infection with COVID-19, abnormal clinical and para-clinical findings were substantially evident [7]. Although the exact mechanisms of liver injury following infection with SARS-CoV-2 is unclear [8], histopathological studies of the liver biopsy in infected patients showed manifestations of probable induced SARS-CoV-2 tissue damages such as moderate microvesicular steatosis and mild lobular and portal activity [9]. Recent studies on COVID-19 have shown that the incidence of liver injury ranged from 14% to 78%, mainly indicated by abnormal ALT/AST levels accompanied by slightly elevated bilirubin levels [4]. Reduction of serum albumin in severe cases is reported too [7]. The severity of progressive liver injury in patients with severe COVID-19 infection was significantly higher than those with mild infection [10].

Although it seemed that our patient had been complicated with a multi-organ failure induced by septic shock following a respiratory infection, the comprehensive evaluation which consisted of cultures and laboratory tests for sepsis workup were against the septic shock.

Heart failure induced hepatopathy is another possibility but the patient's coagulation profile and liver function tests were about normal a week before admission to the ICU. Although high levels of positive end-expiratory pressure can contribute to hepatic congestion by increasing right atrial pressure and impeding venous return, data suggest that many patients admitted to hospital with COVID-19 infection have abnormalities in liver function test [2].

Drug-induced liver injury is another possible contributing factor to the observed abnormal liver function tests in patients with multidrug medications. However, it usually starts after commencing the therapeutic agents and should be considered by clinicians, but same as our patient, liver tests derangement was present at baseline in many patients with COVID-19 infection before dramatic use of accused drugs [11]. In this patient, based on his drug history and recent laboratory tests, his previous medications were not very likely to damage the liver before hospitalization.

Therefore, it seems that the most probable cause of acute liver failure in this patient is neither the drug nor the heart failure, but the occurrence of COVID-19 infection. In other words, although our patient had not shown any obvious liver function derangement or abnormal liver laboratory tests before recent hospitalization, probably the acute infection with COVID-19 caused severe acute liver failure in chronically subclinical damaged liver cells due to long-lasting heart failure. Our case is remarkable because almost all the reports regarding liver complications following COVID-19 infection have denoted mild to moderate liver damage and not so severe as in our case.

## Conclusions

In conclusion, patients with COVID-19 infection, have a varying degree of liver damage and dysfunction; however, consideration of major coexisting diseases such as other chronic organ failure and their effect on liver function is essential. On the other hand, we hope our report of a fulminant hepatic failure related to COVID-19 infection can assist scientists to discover all aspects of the pathogenesis of this recent viral infection, particularly liver failure; as well as it might draw physicians' attention to severe liver involvement that has not been reported frequently.
